# Postoperative pain and short-term complications after two elective sterilization techniques: ovariohysterectomy or ovariectomy in cats

**DOI:** 10.1186/s12917-018-1657-z

**Published:** 2018-11-08

**Authors:** Marco Aurélio A. Pereira, Lucas A. Gonçalves, Marina C. Evangelista, Rosana S. Thurler, Karina D. Campos, Maira R. Formenton, Geni C. F. Patricio, Julia M. Matera, Aline M. Ambrósio, Denise T. Fantoni

**Affiliations:** 1 Faculty of Veterinary Medicine and Animal Sciences, Department of Surgery, University of Sao Paulo, 87, Av Professor Doutor Orlando Marques de Paiva, Sao Paulo, 05508-270 Brazil; 2 Faculty of Veterinary Medicine, Department of Clinical Sciences, Université de Montréal, Saint-Hyacinthe, Quebec, Canada

**Keywords:** Analgesia, Reproductive sterilization, Ovariohysterectomy, Ovariectomy, Cats, Pain

## Abstract

**Background:**

Surgical sterilization of cats is one of the most commonly performed procedures in veterinary practice and it can be accomplished by two different techniques: ovariohysterectomy (OVH) or ovariectomy (OVE). Although there is an apparent preference for OVH in United States and Canada, OVE seems to be the standard of care in many European countries due to its advantages, such as a smaller surgical incision and potentially less complications associated with surgical manipulation of the uterus. The aim of this randomized, blind, prospective study was to compare postoperative pain and short-term complications in cats undergoing ovariohysterectomy or ovariectomy.

**Methods:**

Twenty female cats were randomly assigned into two groups (OVH, *n* = 10 and OVE, n = 10). Pain was assessed prior to surgery (baseline) and 1, 2, 4, 8 12 and 24 h after the procedure using pain and sedation scales, physiologic parameters and blood glucose levels. Short-term complications were evaluated in the early postoperative period and reassessed at day 7 and day 10.

**Results:**

Changes in cardiovascular parameters were not clinically relevant, however cats in OVH group had higher heart rates at T1 h compared with baseline (*p* = 0.0184). Blood glucose levels in OVH group were also higher at T1 h compared with baseline (*p* = 0.0135) and with OVE group (*p* = 0.0218). Surgical time was higher in OVH group (*p* = 0.0115). Even though no significant differences in pain scores were observed between groups or time points, cats in OVH group had greater need for rescue analgesia compared with OVE (2/10 and 0/10, respectively). Complications were not observed in any cat during surgery, at days 7 and 10 postoperatively or at discharge.

**Conclusions:**

Both surgical techniques promoted similar intensity of postoperative pain in cats and there were no short-term complications throughout the study’s evaluation period. Therefore, both techniques may be indicated for surgical sterilization of cats, according to the surgeon’s preference and expertise. Cats that underwent ovariectomy did not require rescue analgesia and surgical time was shorter in that group.

## Background

Elective surgical sterilization of female dogs and cats is one of the most commonly performed procedures in veterinary practice due to its potential benefits, such as population control, prevention of reproductive tract diseases, attenuation of undesirable behaviors associated with hormonal activity and reduction of stray and feral populations of dogs and cats [1].

Sterilization of female cats can be accomplished by surgically removing the ovaries and the uterus (ovariohysterectomy - OVH) or by removing the ovaries only (ovariectomy - OVE). OVH is the most frequently performed technique in some countries like the United States and Canada [1]. Meanwhile, in the Netherlands and some other European countries, OVE is usually performed instead of OVH as the standard of care for sterilization [2]. Furthermore, with the development of minimally invasive surgical techniques, laparoscopic ovariectomy in cats has gained popularity [3–5].

Potential development of uterine diseases and post-surgical complications are important factors to consider in the choice of the sterilization technique. The risk of post-surgical hemorrhage is lower in OVE, partially because this complication is often related to removal of the uterus [6]. Moreover, ovariectomy has several advantages over OVH: it is technically less complicated, it allows shorter surgical and anesthetic time, there is less morbidity due to a smaller incision, less trauma in the abdomen and the broad ligaments are not torn [1, 6, 7]. Regarding postoperative pain in bitches, no significant difference between the two techniques was found [6, 8], however, there are no known studies in cats.

The aim of this study was to assess postoperative pain and short-term complications in cats undergoing either OVH or OVE.

## Methods

### Experimental design

This prospective, randomized, blinded, controlled study was performed at the Veterinary Hospital of the School of Veterinary Medicine and Animal Science, University of Sao Paulo, Brazil.

### Animals

Twenty healthy female domestic cats undergoing OVH or OVE. All animals were deemed healthy (ASA status I) based on physical examination, medical history and laboratory tests (complete blood count, serum chemistry profile and blood glucose). Exclusion criteria included aggressiveness, signs of pre-existing pain or inflammation, underlying diseases and use of analgesic or anti-inflammatory drugs prior to the study. Animals with history of excessive fear when handled were also excluded. Written informed consent was obtained from the owners before inclusion of the animals and the study protocol (8,461,060,715/2016) was approved by the institutional Animal Ethics Committee (AEC).

### Anesthetic procedure

Cats were randomly assigned to two groups (OVH or OVE; *n* = 10 each) with the help of an online random sequence generator (http://www.randomization.com). All cats received acepromazine maleate^1^ (0.1 mg/kg) intramuscularly as premedication. Fifteen minutes after, a 22-gauge catheter was placed in the cephalic vein for fluids (Lactated Ringer’s solution^2^ at 3 mL/kg/h) and drugs administration, then anesthesia was induced with propofol^3^ (5–8 mg/kg). Orotracheal intubation was performed and anesthesia was maintained with isoflurane^4^ diluted in 70% of oxygen in a circular anesthesia circuit. Pressure-controlled mechanical ventilation^5^ was performed (peak inspiratory pressure: 8–10 mmHg, tidal volume: 8 mL/kg and positive end-tidal expiratory pressure: 1 cm H_2_O) and the respiratory rate was adjusted to maintain normocapnia (end tidal carbon dioxide, ETCO_2_: 30–40 mmHg). Remifentanil^6^ constant rate infusion (0.2–0.4 μg/kg/min) was performed to ensure intraoperative analgesia. A single anesthesiologist (LAG) and a single surgeon (JMM) performed all procedures in different dates. Animals were placed on a heating pad with caution to avoid thermal trauma. At the end of the procedure, cats in both groups received meloxicam^7^ (0.1 mg/kg) intravenously at skin closure and 24 h later before hospital discharge.

Heart rate (HR), electrocardiogram (lead II), respiratory rate (RR), oxyhemoglobin saturation, and ventilatory parameters were monitored with a multiparameter bedside monitor^8^; ETCO_2_ and end tidal isoflurane concentration (ET_iso_) were measured by a gas analyzer^9^ and non-invasive systolic blood pressure was measured with the ultrasonic Doppler method.^10^

An increase greater than 15% of baseline values of HR or systolic blood pressure was interpreted as nociception and led to an increase of 0.1 μg/kg/min in remifentanil constant rate infusion.

### Surgical procedures

All surgeries were performed by a single surgeon with more than 25 years of experience (JMM) and one assistant. Standardized surgical protocols were used and the duration of all procedures was recorded, which consisted of the time from the beginning of the skin incision up to its complete closure.

Hair was clipped at the surgical site and skin was aseptically prepared. A ventral midline celiotomy was performed, with its incision starting at the caudal border of the umbilicus. The initial length of the incision was estimated by the surgeon and extended during surgery if necessary. Skin and subcutaneous tissues incision was achieved with an electrosurgical equipment,^11^ which was also used for hemostasis. A small incision was made in the *linea alba* and extended in either direction with scissors to access the abdominal cavity. The left uterine horn was located and the ovary was retracted from the abdomen. Three-clamp technique was used. After section of the ovarian pedicle, an encircling ligature with 4–0 nylon^12^ is placed distal to the two forceps; the ovarian artery and vein were doubly ligated.

In ovariectomy procedures, the ovaries were dissected and excised; the ovarian arteries and veins were ligated with 4–0 nylon^12^ and the uterine horns were returned to their anatomic position. In ovariohysterectomy procedures, after the mesovarium was cut, the broad ligaments were torn parallel to the uterine vessels toward the cervix. The round ligaments were cut with scissors and small vessels were cauterized in case of bleeding. The uterus was removed, and the uterine pedicle was repositioned in its normal anatomic position. All ligated vessels were inspected (in their normal anatomic positions) for bleeding, and used surgical sponges were counted before the abdominal incision was closed.

The abdominal and skin closure were performed with size 4–0 nylon^12^ suture material in a simple interrupted pattern.

### Pain assessment

Prior to the beginning of the study, cats were clinically examined and acclimatized to the observers, cages and sedation and pain assessment tools. Pain was assessed through objective and subjective analysis before premedication (baseline, TBL) and up to 24 h after the end of the surgery, at the following time points: T1 h, T2 h, T4 h, T8 h, T12 h and T24 h.

The objective pain assessment included physiological parameters (HR, RR and rectal temperature – RT) and blood glucose levels.^13^ Subjective pain scores consisted of four pain scales, the Visual Analogue Scales – VAS and three other scales described for the feline species (The Colorado State University Feline Acute Pain Scale - Colorado, The UNESP-Botucatu multidimensional composite pain scale for assessing postoperative pain in cats – UNESP, The Glasgow Feline Composite Measure Pain Scale – Glasgow) [9–12]. Sedation was assessed with a scale adapted from Valverde et al. (2004) [13]. Rescue analgesia (tramadol hydrochloride,^14^ 2 mg/kg intravenously) was administered if the cat reached a score greater than or equal to the cut-off value for rescue in any scale (VAS ≥ 4, Colorado ≥2, UNESP ≥7, Glasgow ≥5). The person responsible for pain assessment (MAAP) was unaware of the surgical technique performed.

Meloxicam 0.1 mg/kg (PO, q 24 h for 1 day) and tramadol 2 mg/kg (PO, q 12 h for 3 days) were prescribed at hospital discharge.

### Postoperative complications

The occurrence of adverse effects such as intra-abdominal hemorrhage during and after surgery, erythema, swelling, vaginal discharge, urinary incontinence, suture dehiscence and signs of infections, was investigated in the early postoperative period (up to 24 h after surgery) and reassessed at day 7 (follow up for complete blood count and serum chemistry) and day 10 (suture removal) of the postoperative period. The owners were contacted daily (by telephone) and asked about the cats’ general condition (appetite, comfort), presence of abnormal behaviors and general appearance of the surgical wound.

### Statistical analysis

Statistical analysis was performed using a statistical software.^15^ Significance was defined as *p* < 0.05 (two-tailed). Results are presented as mean ± standard deviation (SD). Weight and surgical time were compared between groups by unpaired t-tests. For physiological parameters and blood glucose levels, two way-ANOVA and Sidak’s post-test were performed to compare groups and time points. Differences between baseline and other time points’ values in each group were assessed by Dunnett’s post-test. For nonparametric data, such as pain and sedation scores, Krukal-Wallis test and Dunn post-test were used to compare groups throughout time, and the Friedman test followed by Dunn post-test was used to identify differences within a group. Fisher’s exact test was used to analyze the number of rescue analgesia interventions.

## Results

Twenty-two cats were initially selected, however two had to be excluded due to early pregnancy (*n* = 1) and mesenteric lymphadenitis (n = 1). Finally, 20 healthy female cats, without gross reproductive abnormalities were included in this study; OVH group (*n* = 10) and OVE group (n = 10).

Body weight (mean ± SD – OHE: 2.71 ± 0.49 kg, OVE: 2.88 ± 0.58 kg; *p* = 0.5032) and age (OHE - 15 ± 13 months, OVE – 11 ± 6 months; *p* = 0.4327) were not different between groups.

Surgical time differed significantly between groups. The OVH technique was longer than OVE (30 ± 4 min and 25 ± 5 min, respectively, *p* = 0.0115).

### Physiological parameters

Baseline parameters were similar between groups. Respiratory rate did not differ between groups, or among time points observed. Heart rate was higher in OVH than in OVE group at 1 h (*p* = 0,0184). Rectal temperature decreased in both groups at T1 h, compared to baseline. Blood glucose levels were higher in OVH compared to OVE (*p* = 0.0218) and to baseline values (*p* = 0,0135) at 1 h. (Table 1).

**Table 1:** Physiological parameters and blood glucose of cats undergoing ovariohysterectomy (OVH) or ovariectomy (OVE) at baseline and up to 24 h postoperatively

		Baseline	T_1h_	T_2h_	T_4h_	T_8h_	T_12h_	T_24h_
HR (bpm)	OVH	208 ± 28	241 ± 22^*^	215 ± 26	207 ± 17	206 ± 26	202 ± 20	205 ± 29
	OVE	214 ± 42	238 ± 23	215 ± 20	210 ± 28	205 ± 28	200 ± 13	196 ± 15
RR (mpm)	OVH	56 ± 19	52 ± 26	48 ± 13	52 ± 20	54 ± 12	52 ± 14	56 ± 12
	OVE	56 ± 13	53 ± 12	47 ± 12	48 ± 12	51 ± 11	47 ± 11	46 ± 11
RT (°C)	OVH	38,5 ± 0	36,6 ± 1*	37,8 ± 1	38,6 ± 1	38,3 ± 1	37,9 ± 0	37,8 ± 0
	OVE	38,3 ± 0	36,5 ± 1*	37,7 ± 1	38,2 ± 1	38,3 ± 1	38,3 ± 1	38,2 ± 1
Blood glucose (mg/dL)	OVH	78 ± 16	103 ± 34* ^b^	95 ± 22	92 ± 14	84 ± 7	81 ± 9	79 ± 12
	OVE	77 ± 9	79 ± 21 ^a^	85 ± 24	98 ± 17	76 ± 8	73 ± 14	73 ± 10

Data are expressed as mean ± SD. *HR* Heart rate expressed in beats per minute; *RR* Respiratory rate expressed in movements per minute; *RT* Rectal temperature in Celsius; *Value significantly different (*p* < 0.05) from TBL for that group. Letters means values significantly different among the groups in each time point, as b > a

### Pain assessment

Sedation scores both in OVH and OVE groups were higher than baseline at 1 h (*p* < 0.0001 and *p* = 0,0014, respectively).

**Fig. 1:**
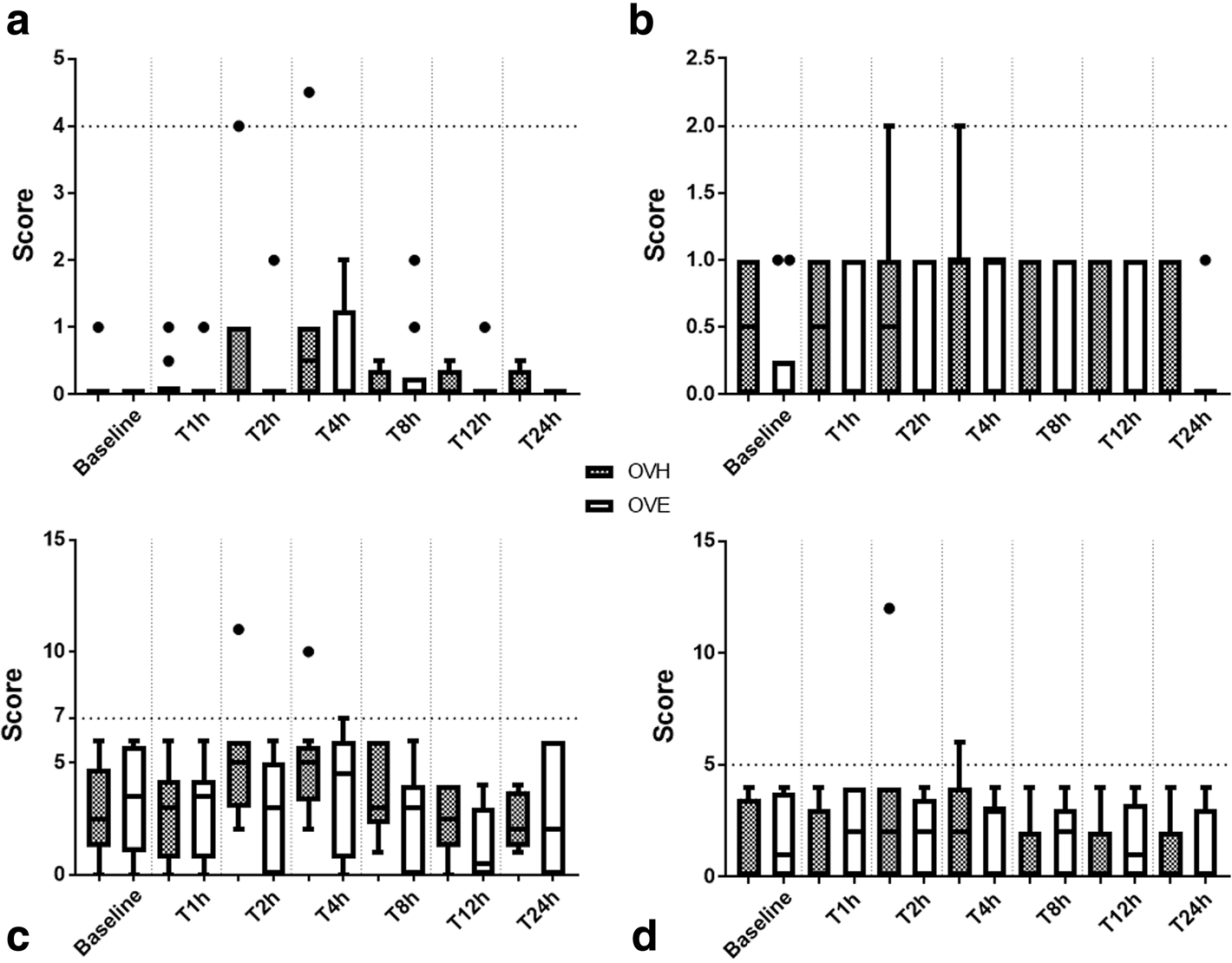
Box plot showing median, interquartile range, minimum and maximum pain scores according to the subjective pain scales during the study. Differences between groups or time points compared with baseline were not observed in the Visual Analogue Scale (**a**), Colorado state university feline acute pain scale (**b**), UNESP-Botucatu multidimensional composite pain scale for assessing postoperative pain in cats (**c**) and Glasgow feline composite measure pain scale (**d**)

Although no significant difference was observed between groups or among time points, OVH group had higher pain scores and greater overall need for rescue analgesia. (Fig. 1).

In the OVH group, 2/10 cats required rescue analgesia. One cat received tramadol at T2 h (Scores: VAS = 4, Colorado = 2, UNESP = 11 and Glasgow = 12) and the other received it at T4 h (Scores: VAS = 4.5, Colorado = 2, UNESP = 10 and Glasgow = 6). No cats required additional analgesia in OVE group.

### Short-term complications

Owners did not notice any changes in the cats’ behavior or in the general appearance of the surgical wound. No complications were observed during the follow-up consultations at day 7 and day 10.

## Discussion

Both surgical sterilization techniques produced similar postoperative pain levels and there were no short-term complications throughout the study’s period of evaluation. This clinical study mimics the setting of veterinary practice, where young, healthy female cats are commonly spayed. A standardized anesthetic protocol and a single surgeon were chosen to reduce potential bias in the intraoperative period and postoperative pain assessments. Anesthetic and surgical complications were not observed.

Corroborating the results of previous studies performed in bitches [2, 8], surgical time differed between groups. Duration of the procedures was significantly longer in OVH than in OVE. This result confirms ovariectomy’s potential advantage.

The choice of remifentanil as part of the anesthetic protocol was based on its short half-life and minimal residual analgesic effect, so that it does not interfere in postoperative sedation and analgesia [14]. Acepromazine was administered as premedication in all animals. Its antinociceptive effect was studied previously in cats and increased pressure nociceptive thresholds were observed up to 2 h [15]. In the present study, 90% of the animals showed sedation scores of 0 at T2 h, including those who needed rescue analgesia at this time point.

Changes in physiologic parameters, despite significantly different, were not clinically relevant. These changes may have been influenced by several factors other than pain, such as environmental conditions, stress associated with manipulation and behavior of the animal [16, 17]. Consequently, HR alone is a poor indicator of pain in cats [16–21]. Nevertheless, one of the cats showed increased HR and blood glucose levels in the moment it required rescue analgesia, which could be associated with pain.

Rectal temperature decreased in all groups at T1 h when compared with TBL. This is justified by the central nervous system depression caused by the inhalation anesthetic (isoflurane), which decreases the sensitivity of the thermoregulatory center of the hypothalamus, and also by the fact that small animals (under 5 kg) have higher risk of developing hypothermia due to their larger surface area to volume ratio [22]. Moreover, acepromazine is known to cause mild hypotension due to vasodilation, which contributes to decreasing body temperature [15]. An attempt to attenuate hypothermia was performed by placing the animals on a heating pad during surgery.

In the first postoperative assessment time point (T1 h), blood glucose levels were significantly higher in the OVH group compared both with its baseline values and with OVE group. That could have been caused by the substantial increase observed in one cat that required analgesic rescue (T2 h). This cat’s blood glucose levels at baseline, T1 h and T2 h were 73, 164 and 130 mg/dL, respectively. Pain, as a stress factor, may be associated with increases in blood glucose concentration. Changes in blood glucose levels were previously described as part of analgesic efficacy assessment [23, 24].

Pain scores were not significantly different between groups or compared with baseline values. Similar findings have been previously observed in bitches undergoing OVH or OVE [6, 8]. In the present study, four subjective pain measurement tools [9–12] were used to further quantify differences after surgery, however no statistical differences between groups was found. This may be explained by the fact that the analgesic regimen used during and after surgery was satisfactory for both surgical techniques, thus the pain experienced by the cats was only mild.

The subjective nature of pain assessment and the difficulty in recognizing behavioral signs that may be indicative of painful states, especially in a hospital environment, are some of the inherent limitations of pain scales. A study that investigated the correlation between the UNESP-Botucatu and the CMPS-Feline pain scales have shown strong association between the tools, however outcome for rescue analgesia would differ when using each of scales [25]. In the present study, all cats showed scores above the cut-off values of all scales in the moments when they required rescue analgesia.

The Portuguese version (native language of the evaluators) of the UNESP-Botucatu scale was used. The Colorado and Glasgow pain scales were used in their original language of publication (English). The observer’s level of experience and cultural and language differences are other factors that might influence pain assessment using scales [26]. Due to the difficulty in measuring blood pressure in non-anesthetized or sedated cats in a reliable way [12, 27] we did not use this part of the UNESP-Botucatu scale (subscale 3 - physiological variables). This scale has been previously validated to be used not only in its full version, but also considering each of the subscales, so that they could be independently evaluated and even omitted if necessary. Considering the descriptive and ROC curve analysis, rescue analgesia should be performed if scores reach at least 26.6% of the scale’s maximum score and is strongly recommended if it reaches at least 33.3% [12]. Therefore, 7/27 in the UNESP-Botucatu pain scale was defined as the cut-off value for the administration of rescue analgesia.

The anesthetic and analgesic protocols provided adequate analgesia in the postoperative period in most of the cats. Two cats in OVH group required rescue analgesia, while there was no need for additional analgesia in the OVE group. This could be related to the greater degree of trauma in the abdomen and to the rupture of the broad ligaments that occur during ovariohysterectomy, which may correspond to a higher level of nociception [8, 28] and might have contributed to the need for rescue analgesia in OVH group. The administration of tramadol as rescue analgesia controlled pain adequately.

One of the limitations of the present study is that abdominal echography was not performed in the perioperative period. We could only evaluate the complications based on expected clinical signs or behavioral changes based on owners’ report.

## Conclusions

Both OVH and OVE promoted similar intensity of postoperative pain and absence of surgical complications in cats. Either technique may be indicated for surgical sterilization, according to the surgeon’s preference and expertise. Although no significant differences were observed in pain scores between groups, two cats in OVH group required rescue analgesia, whereas none required it in OVE group, and surgical time was shorter in OVE procedure. No short-term complications were observed throughout the study’s evaluation period.

## Abbreviations

HR: Heart rate
OVE: Ovariectomy
OVH: Ovariohysterectomy
RR: Respiratory rate
RT: Rectal temperature

## Footnotes

1 Acepran 0,2%, Vetnil, SP, Brazil.

2 Ringer Lactato, Baxter Hospitalar Ltda., SP, Brazil

3 Propovan, Cristália, SP, Brazil

4 Isoflorane, Cristália, SP, Brazil

5 Anesthetic Machine Inter Linea A, Intermed, SP, Brazil

6 Ultiva 2 mg, GlaxoSmithKline, RJ, Brazil

7 Maxicam 0,2%, Ourofino Saúde Animal, SP - licensed for IV use in Brazil

8 Multi-parameter Patient Monitor DX 2020, Dixtal Biomédica Ind. Com. Ltda., SP, Brazil

9 Anesthetic Gas Monitor The Poet IQ2, Critcare Systems, Inc., North Kingstown, U.S

10 Ultrasonic Doppler Flow Detector Model 812, Parks Medical Eletronics, Inc., Oregon, U.S

11 Aspen Excalibur Plus PC Electrosurgical Unit, CONMED, New Jersey, U.S

12 Mononylon Ethilon 4.0, Ethicon US, LLC, U.S

13 Glycosimeter Optium Xceed™, Abbott Laboratories**,** Philippines

14 Cronidor 2%, Agener União Saúde Animal, SP, Brazil

15 GraphPad Prism 7.03 Software Inc., La Jolla, U.S
